# Remotely sensed measures of Hurricane Michael damage and adverse perinatal outcomes and access to prenatal care services in the Florida panhandle

**DOI:** 10.1186/s12940-022-00924-1

**Published:** 2022-11-29

**Authors:** Ke Pan, Elaina Gonsoroski, Christopher K. Uejio, Leslie Beitsch, Samendra P. Sherchan, Maureen Y. Lichtveld, Emily W. Harville

**Affiliations:** 1 Department of Epidemiology, School of Public Health and Tropical Medicine, Tulane University, Tidewater 1820, 1440 Canal St, New Orleans, LA 70112 USA; 2 Department of Geography, College of Social Sciences and Public Policy, Florida State University, Tallahassee, FL 32306 USA; 3 Department of Behavioral Sciences and Social Medicine, College of Medicine, Florida State University, Tallahassee, FL 32306 USA; 4 Department of Biology, Morgan State University, Baltimore, MD 21251 USA; 5 Department of Environmental and Occupational Health, School of Public Health, University of Pittsburgh, Pittsburgh, PA 15261 USA

**Keywords:** Disaster, Hurricane, Remote sensing, Prenatal care, Pregnancy, Birth

## Abstract

**Background:**

Studies of effects of hurricanes on perinatal outcomes often rely on approximate measures of exposure. This study aims to use observed damage from aerial imagery to refine residential building damage estimates, evaluate the population changes post landfall, and assess the associations between the extent of residential building damage and adverse perinatal outcomes and access to prenatal care (PNC) services.

**Methods:**

Vital statistics data from the Florida Department of Health’s Office of Vital Statistics were used to align maternal geocoded address data to high-resolution imagery (0.5-foot resolution, true color with red, blue, and green bands) aerial photographs. Machine learning (support vector machines) classified residential roof damage across the study area. Perinatal outcomes were compared with the presence or absence of damage to the mother’s home. Log-binomial regression models were used to compare the populations living in and outside of high-risk/damage areas, to assess the population changes after Hurricane Michael, and to estimate the associations between damage after Hurricane Michael and adverse perinatal outcomes/access to PNC services. A semi-parametric linear model was used to model time of first PNC visit and increase in damage.

**Results:**

We included 8,965 women in analysis. Women with lower education and/or of Black or other non-White race/ethnicity were more likely to live in areas that would see high damage than other groups. Moreover, there was a greater proportion of births delivered by women living in the high-risk/damage area (> 25% damaged parcels after Michael) in the year before Michael than the year after Michael. Lastly, living in the area with relatively high damage increased the risk of having intermediate or inadequate PNC (adjusted Risk Ratio = 1.21, 95% CI: 1.03, 1.43), but not other adverse perinatal outcomes.

**Conclusions:**

Aerially observed damage data enable us to evaluate the impact of natural disasters on perinatal outcomes and access to PNC services based on residential building damage immediately surrounding a household. The association between the extent of damage and adverse perinatal outcomes should be further investigated in future studies.

**Supplementary Information:**

The online version contains supplementary material available at 10.1186/s12940-022-00924-1.

## Background

Disasters are associated with an increased risk of inadequate prenatal care (PNC) and adverse perinatal outcomes, including cesarean section, lower birth weight (LBW), and smaller gestational age (SGA) at birth, preterm birth (PTB), and Fetal Distress [1–8]. In most previous studies, exposure to disasters has been indicated by region of residence and timing of birth, and the effects of disasters were assessed by comparing births before and after disasters only [1, 7–9]. For example, comparing births occurring two years after Hurricane Katrina to births two years before, the rates of cesarean section and inadequate PNC rose in the most affected areas in Louisiana (cesarean section: adjusted OR (aOR) = 1.13, 95% confidence interval (CI): 1.09–1.18; inadequate PNC: aOR = 1.10, 95%CI: 1.07–1.12) [1]. Another study compared pregnancy outcomes before and after the Hurricane Harvey in Houston Texas in August 2017. The composite maternal morbidity and neonatal morbidity increased by 27% (aOR = 1.27, 95%CI: 1,14–1,42) and 50% (aOR = 1.52, 95%CI: 1.34–1.71), respectively, after Hurricane Harvey compared to before [9]. The effect of Hurricane Harvey on perinatal outcomes were also assessed in southern Florida, Louisiana, and Mississippi. The study found that maternal exposure to Hurricane Harvey in second and third trimesters was associated with an increased risk of fetal distress [7]. While suggestive, these studies are subject to potential ecologic bias (participants who suffered adverse outcomes may not be the ones exposed to the disaster) as well as exposure measurement error (disaster effects vary widely within an area as large as a county).

Remotely sensed data have been used in the assessment of damage situations after natural disasters [10], and have the potential to ameliorate some of these shortcomings. Damage extent of individual buildings, such as damage to roofs caused by wind pressure, can be detected by using high-resolution aerial or satellite images. Assessing damage after natural disasters in this way can leverage capabilities and expertise to provide support for response and recovery efforts [11]. Moreover, remotely sensed data can be used to determine the exposure of population to natural disasters and evaluate its effects on health outcomes [12–14]. For example, satellite-based data were used to measure the association between flooding and emergency department visits after Hurricane Harvey [12]. By measuring individual-level residential property damage after disasters and comparing the risks of adverse health outcomes across populations exposed to different levels of damage, this approach might provide more accurate “dose–response” estimates of the effects of disasters, such as hurricanes, rather than simply comparing health outcomes before and after disasters.

Such data may also be valuable for assessing population effects of disasters. Disasters, natural and technological, disproportionately harm minorities and low-income residents [1], perhaps due to residential patterns surrounding disaster risk. For example, minority and low-income residents were more likely to live in high flood risk areas in South Florida, possibly because minorities and low-income residents may be less likely to afford homes outside the flood zone [15].

The effects of disasters can also have large impacts on the population and migration, which can have lasting impacts on communities. For instance, compared to one year before Hurricane Katrina, a natural and technological disaster, there were 4199 fewer births overall in Louisiana and 6468 fewer births in the most affected region in one year after Katrina [1]. Similarly, approximately 2–4% of the population, especially those of school- or working age, left Puerto Rico in the year after Hurricane Maria [16].

In October 2018, Hurricane Michael, a category 5 hurricane targeting the largely rural Florida Panhandle, caused extensive structural damage and widespread power outages. In this study, we used high resolution aerial imagery to classify residential building damage after Hurricane Michael to (a) compare the populations giving birth and living in and outside of high-risk/damage areas before and after the hurricane, respectively; (b) evaluate the changes in characteristics of population giving birth after Hurricane Michael; (c) assess whether the extent of damage after Hurricane Michael was associated with change in risk of adverse perinatal outcomes and access to PNC services.

## Methods

### Study population

We obtained 2017–2019 vital statistics data (birth certificate) from the Florida Department of Health’s Office of Vital Statistics. Eligible births were based on FEMA disaster declarations [17] to mothers whose county of residence received public and individual assistance (most affected areas). The analysis covered births delivered in the year before and after Hurricane Michael (before: Oct.6^th^, 2017 to Oct. 5^th^; after: Oct. 6^th^ to Oct. 6^th^, 2019). Supplementary Table 1 and Supplementary Fig. 1 shows the numbers of births in each county.

### Damage assessment

We used high resolution aerial imagery to estimate residential building damage in the affected counties. The imagery is available from the Land Boundary Information System which is sponsored by The Florida Department of Environmental Protection (FDEP), Division of State Lands, Bureau of Survey and Mapping. Images were available for 11 of the 12 counties designated for both individual and public assistance by FEMA after Hurricane Michael. The only county without images available was Leon County.

**Fig. 1:**
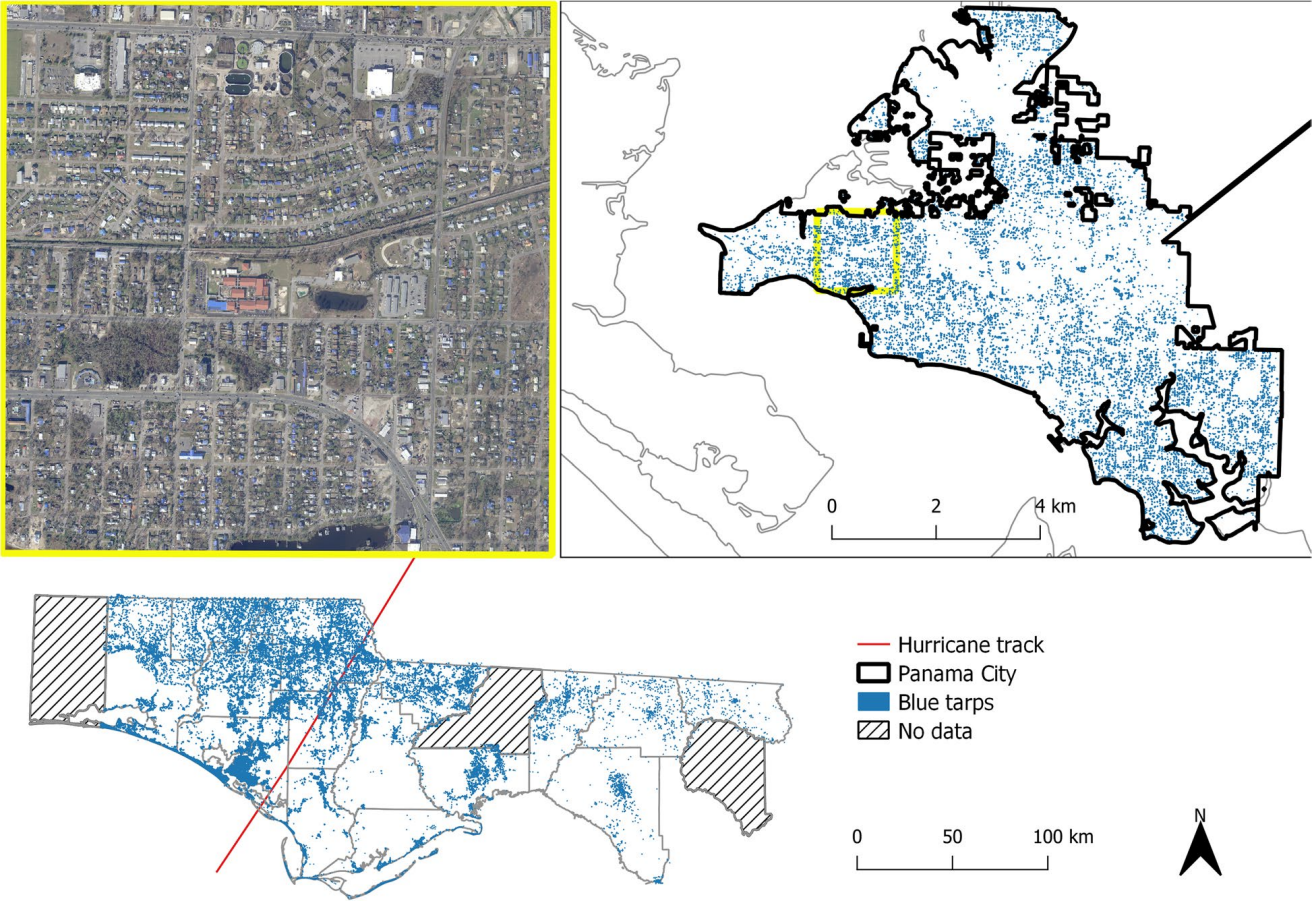
The aerial image (top left) shows an urban area with a number of roofs covered by blue tarps. *The image covers one square mile in Panama City, FL (top right). The blue tarps in the study area are more densely distributed near the hurricane track but were detected throughout much of the study area (bottom)

The aerial images contained a red, blue, and green visible color bands and 0.5 × 0.5 ft spatial resolution. Figure 1 shows the tarp distribution in the study area, a focus in one populated area (Panama City, FL), and a true color aerial image which covers one square mile within Panama City, FL. Features reflect visible light differently which can be used to distinguish objects on the ground in the images. The images were collected 2–3 months after Hurricane Michael’s landfall. We used blue tarps as an indicator for building damage. Based on the availability of high resolution aerial imagery, the analysis presumes that roofs were undamaged/not covered with blue tarps prior to Hurricane Michael’s landfall. Previous studies have demonstrated the use of blue tarps as post-hurricane indicators of damage and recovery, and the tarps are distinguishable using optical imagery [18, 19]. The data were classified in Python 3 with the sci-kit learn package [20]. A Support Vector Machine model classified the images first as a multiclass classification with seven classes: blue tarp, pool water, impervious surface, vegetation, bare soil, roof, and natural waterbody. This was then simplified to a binary classification (tarp or no tarp present). The model had an overall accuracy of 85.3% with a sensitivity of 74% and a specificity of 96.7%.

We complemented the aerial imagery with a publicly available “windshield” damage assessment in Leon County, which contains the state capital (Tallahassee) and one of the larger affected populations. Hurricane Michael caused notable damage in Leon County (which qualified for individual FEMA assistance) but was less catastrophic there than counties located closer to where the storm made landfall [21]. The county experienced 5851 births, which was 39.49% of the total study area births. Leon County completed a rapid, vehicle based “windshield” damage assessment within three days of the hurricane’s landfall. Damage assessors drove across the county and documented damage with photos and completed a standardized damage survey in ArcGIS collector.

The Florida Vital Statistics Office included geocoded maternal addresses with the requested records. Data were stored in secure, password-protected files on central servers at Tulane and FSU and accessed through encrypted computers. All staff with access to data received training in human subjects research, including confidentiality and data protection. We used tax parcel data from the University of Florida’s GeoPlan Center to match the geocoded births to tarp presence in a Geographic Information System. Damaged parcels were defined as a residential property with a blue tarp > 18.6 m^2 while all other properties were considered undamaged. However, the birth record geocoding process contained geographic positional error where addresses did not directly overlap with household property boundaries. Previous studies have demonstrated that geocoding positional error varies by method used and whether the location is in an urban or rural setting. However, for the majority of cases, the error is less than 100 m with an average of approximately 50 m [22]. To estimate infrastructure damage exposure more accurately, we used the proportion of parcels around a geocoded birth that had blue tarp present. We created 25 m, 50 m, and 100 m buffers around the geocoded birth record location and calculated the percentage of blue tarp damaged parcels.

### Outcomes-perinatal outcomes and access to PNC services

Perinatal outcomes and access to PNC services were assessed using vital statistics. Perinatal outcomes include LBW, preterm birth (PTB), SGA, Caesarean section, breastfeeding, and access to PNC services. LBW was defined as a birthweight of an infant of 2,500 g or less, regardless of gestational age. PTB was defined as a birth before 37 weeks of gestation. Based on national standards [23], SGA was defined by birthweight below the 10th percentile for gestational age. Mode of delivery was defined as Caesarean section vs. other methods. Breastfeeding was based on the indicator for infants being breastfed between birth and discharge. Access to PNC services were evaluated by whether pregnant women had any PNC visits before delivery, the month of first PNC visit, and the Kotelchuck Index. Kotelchuck Index used information (PNC initiation time and number of PNC visits) from birth certificate to assess the PNC utilization adequacy. There are four adequacy categories in the Kotelchuck Index: adequate plus, adequate, intermediate, and inadequate [24].

### Covariates

Known risk factors from the Vital Statistics for outcomes were assessed as potential confounders, including maternal age, race, ethnicity, education, smoking during pregnancy, alcohol use, pre-pregnancy BMI, and whether enrolled in the U.S. Department of Agriculture’s Supplemental Nutrition Program for Women, Infants, and Children (WIC) program. A causal diagram was created to identify confounders for each association we assessed [25]. The confounders were selected if these represented risk factors for the outcome of interest, were associated with the exposure (damage) but were not intermediate variables in the causal pathway between exposure and outcome.

### Statistical analyses

Complete case analysis was conducted to assess the association between the percent of parcels damaged within a given radius and perinatal outcomes and access to PNC services. Outcome and covariate missing data were minimal. Missing data were categorized as follows: LBW (0.19%), preterm (0%), SGA (0%), time of first PNC visit (13.01%), PNC overall use (0.48%), Kotelchuck Index (16.74%); maternal age (0%), education (2.98%), race/ethnicity (1.80%), pre-pregnancy BMI (7.55%), WIC enrollment (1.77%), smoking during pregnancy (0.88%), and alcohol drinking during pregnancy (6.64%).

**Fig. 2:**
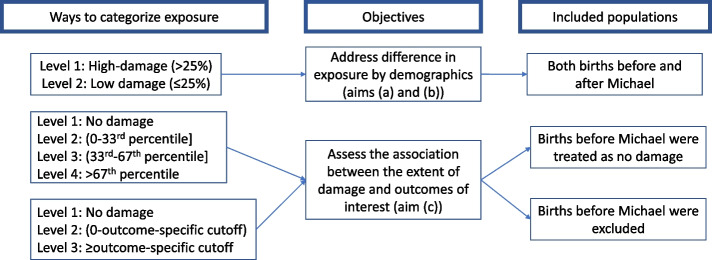
Analytic strategy

Exposure was categorized in three ways for the purpose of presenting demographics data and exploring different thresholds as we explored a new method to measure associations between hurricane and pregnancy outcomes (Fig. 2): First, it was dichotomized into areas of high (> 25% of parcels within a 25 m radius of the woman’s residence) and low damage (≤ 25%) to present the demographics of women giving birth before and after Hurricane Michael in the most affected areas. Because damages were measured after Hurricane Michael only, those areas were defined as areas of high (> 25%) and low (≤ 25%) risk for births happened before Hurricane Michael. Secondly, we further categorized the percent of parcels damaged into four categories, no damage, <  = 33rd percentile, 33rd-67th percentile, > 67^th^ percentile, using a radius of 25 m. Sensitivity analysis repeated the aforementioned analyses using a radius of 50 m and 100 m (Supplementary Table 2). Lastly, threshold regression was used to decide outcome-specific cutoffs of exposure (using the percent of parcels damaged within a 25 m radius) for each outcome of interest. Threshold models iteratively tested potential breaks points identify cutoffs of the proportion of blue tarp through Bayesian information criteria (BIC), Akaike information criterion (AIC), or Hannnan-Quinn information criterion (HQIC) [26], choosing the best cut-off to maximize model fit. The damage variable was categorized into three categories: 0%, 0% ~ outcome-specific cutoff, >  = outcome-specific cutoff. Two sets of analyses were performed to assess the association between the extent of residential building damage and perinatal outcomes/access to PNC services. In the first scenario, births in the year before Hurricane Michael were coded as zero (no damage). In the second scenario, all births before Hurricane Michael were excluded.

Log-binomial regression models were used to compare the populations giving birth and living in and outside of high-risk/damage areas before and after the hurricane, respectively. Interaction terms between hurricane (before and after) and high-risk/damage areas (> 25% damage) in log-binomial regression models were used to evaluate the changes in population giving birth after Hurricane Michael. Log-binomial regression was used to estimate the associations between damage after Michael and binary outcomes (whether developed LBW, PTB, SGA, or had Caesarean section, breastfeeding, any PNC visits, intermediate/inadequate PNC). A semi-parametric linear model was used to evaluate the association between damage and time of first PNC visit. All estimates were compared unadjusted and after adjusting for potential confounders. Model performance was assessed by using the Value/DF of deviance. The Value/DF of deviance for models above ranged from 0.85 to 1.25. The main analyses (presented in the main text) (included 11 counties) excluded Leon County to have a consistent measure of damage from aerial imagery. Sensitivity analyses included Leon County’s complementary windshield survey (Supplementary Tables 3 and 4). Threshold regression was conducted in the software Stata 15 (College Station, TX: StataCorp LLC), and other statistical analyses were performed using the software SAS 9.4 (SAS Inc., Cary, NC).

This analysis was approved by the Institutional Review Boards of Tulane University (2019–529-TUHSC), Florida State University, and the Florida Department of Health.

## Results

We first examined whether the population giving birth lived in areas that were damaged by the hurricane. In order to assess the extent of changes in population giving birth due to the Hurricane, we examined the areas where women giving birth lived prior to the hurricane and determined whether they were damaged. Examining changes in demographic factors in affected areas indicates whether certain groups were more likely to experience severe damage (were more vulnerable to the hurricane). Therefore, 8,965 births from October 6, 2017 to October 6, 2019 in 11 of the 12 counties of residence received public and individual assistance ((most affected areas). were categorized as occurring to mothers living in areas which had <  = 25% or > 25% damage after Hurricane Michael. To determine if the number of births went down in damaged areas after the hurricane, we calculated the median proportion of households damaged at three distances (25 m, 50 m, and 100 m) from the geocoded births which occurred before and after the hurricane. For geocoded births that occurred after Michael, the median proportion of damaged parcels within a 25 m, 50 m, and 100 m radius was 0% (Q1-Q3: 0–25%), 5.56% (Q1-Q3: 0–25%), and 8.70% (Q1-Q3: 0–21.74%) respectively. To look at it another way, there were 4678 and 4287 births in the year before and after Michael, respectively. The difference of number of births in the year before and after Michael was not statistically significant (*p* = 0.38). However, in areas having high damage (> 25% damaged parcels) after Hurricane Michael, there were significantly more women who had births before Michael compared to the year after Michael (1258 (26.89%) vs. 998 (23.28%), *p* < 0.0001).

The study population giving birth in different areas before and after Hurricane Michael is described in Table 1, respectively. Compared with women living in areas with low damage (≤ 25% damaged parcels), there were more women who received the Supplemental Nutrition Program for Women, Infants, and Children (WIC) services and who did not have a college degree or higher living in areas with high damage before and after Michael. In Table 2, we compared the population giving birth and living in area with high damage before and after Hurricane Michael. The percentages were calculated as the proportions of women living in areas with high damage in one category (such as women with high school education or GED or less). In the year before the hurricane, 22% of women with a college degree or higher lived in areas with high damage in the hurricane, compared to 30% of those with some college or a technical degree, and 26% of those with a high school degree or lower. After the hurricane, beyond the overall tendency for fewer births in the most damaged areas, the decline was greatest among those with some college or technical degree (risk difference(RD) = -6.2%, 95%CI: -2.88%, -9.56%) and Hispanic White women (RD = -5.0%, 95%CI: -2.48%, -12.48%) and lowest among those with high school or GED degree or less (RD = -1.5%, 95%CI: -4.09%, 1.02%).

**Table 1:** Demographics of women giving birth before and after Hurricane Michael in the most affected areas in Florida (2017–2019)

	**Before**			**After**		
	**Low risk (25 m)**	**High risk (25 m)**	***p*-value**	**Low damage (25 m)**	**High damage (25 m)**	*p***-value**
	**Mean (SE)/ N (%)**			**Mean (SE)/ N (%)**		
**Total number of births**	3420	1258		3289	998	
**Maternal age**	27.23 (5.69)	26.91 (5.56)	0.08	27.31 (5.70)	27.07 (5.96)	0.24
**Maternal education level**						
High School or GED or less	1699 (50.72)	604 (49.51)	< 0.01	1641 (51.75)	538 (56.22)	< 0.01
Some College Credit, but No Degree or Associate Degree	1063 (31.73)	452 (37.05)		938 (29.58)	290 (30.30)	
Bachelor’s Degree and above	588 (17.55)	164 (13.44)		592 (18.67)	129 (13.48)	
**Maternal ethnicity**						
Non-Hispanic White	2323 (68.67)	814 (66.23)	0.06	2235 (69.11)	619 (64.61)	< 0.01
Hispanic White	189 (5.59)	65 (5.29)		189 (5.84)	49 (5.11)	
Black	701 (20.72)	264 (21.48)		672 (20.78)	222 (23.17)	
Other	170 (5.03)	86 (7)		138 (4.27)	68 (7.10)	
**WIC program**	1980 (59.16)	792 (64.71)	< 0.01	1761 (54.02)	576 (59.08)	< 0.01
**Pre-pregnancy BMI**	27.91 (7.25)	28.23 (7.66)	0.22	28.20 (7.36)	28.47 (8.02)	0.36
**Smoking during pregnancy**	278 (8.18)	103 (8.22)	0.96	354 (10.88)	101 (10.31)	0.61
**Alcohol use during pregnancy**	36 (1.12)	6 (0.49)	0.05	24 (0.79)	3 (0.33)	0.13
**Low birth weight (LBW)**	245 (7.17)	105 (8.37)	0.17	296 (9.02)	92 (9.23)	0.84
**Preterm birth (PTB)**	431 (12.60)	161 (12.80)	0.86	403 (12.25)	104 (10.42)	0.12
**Small for gestational age (SGA)**	387 (11.32)	150 (11.92)	0.56	434 (13.20)	130 (13.03)	0.89
**Caesarean section**	1095 (32.03)	382 (30.41)	0.29	1079 (32.82)	317 (31.80)	0.55
**No breastfeeding**	776 (23.21)	251 (20.51)	0.05	714 (22.06)	239 (24.72)	0.08
**Gestational month of 1st PNC**	2.81 (1.57)	3.03 (1.77)	0.002	2.98 (1.74)	3.11 (1.90)	0.16
**PNC (yes/no)**	3356 (98.79)	1237 (99.20)	0.23	3218 (98.11)	977 (97.90)	0.67
**Intermediate/Inadequate PNC**	708 (24.82)	281 (27.79)	0.06	866 (31.03)	278 (34.36)	0.07

*GED* General Educational Development, *WIC* Women’s, Infant’s, and Children, *BMI* body mass index

**Table 2:** Demographics of women giving birth in areas with high damage before and after Hurricane Michael in the most affected areas in Florida (2017–2019)

	**High risk/damage area**			
	**Before**	**After**	**Difference**	***p*-value**
	**Mean (SE)/ N (%)**			
**Total number of births**	1258 (26.89)	998 (23.28)	-3.61%	< 0.01
**Maternal age**	26.91 (5.56)	27.07 (5.96)	0.16	0.77
**Maternal education level**				
High School or GED or less	604 (26.23)	538 (24.69)	-1.54%	0.02
Some College Credit, but No Degree or Associate Degree	452 (29.83)	290 (23.62)	-6.22%	0.05
Bachelor’s Degree and above	164 (21.81)	129 (17.89)	-3.92%	0.56
**Maternal ethnicity**				
Non-Hispanic White	814 (25.95)	619 (21.69)	-4.26%	0.37
Hispanic White	65 (25.59)	49 (20.59)	-5.00%	0.71
Black	264 (27.36)	222 (24.83)	-2.53%	0.42
Other	86 (33.59)	68 (33.01)	-0.58%	0.44
**WIC program**	792 (28.57)	576 (24.65)	-3.92%	0.84
**Pre-pregnancy BMI**	28.23 (7.66)	28.47 (8.02)	0.36	0.90
**Smoking during pregnancy**	103 (27.03)	101 (22.20)	-4.83%	0.67
**Alcohol use during pregnancy**	6 (14.29)	3 (11.11)	-3.18%	0.73
**Low birth weight (LBW)**	105 (30)	92 (23.71)	-6.29%	0.46
**Preterm birth (PTB)**	161 (27.20)	104 (20.51)	-6.69%	0.21
**Small for gestational age (SGA)**	150 (27.93)	130 (23.05)	-4.88%	0.63
**Caesarean section**	382 (25.86)	317 (22.71)	-3.15%	0.80
**No breastfeeding**	251 (24.44)	239 (25.08)	0.64%	0.01
**Gestational month of 1st PNC**	3.03 (1.77)	3.11 (1.90)	0.08	0.30
**PNC (yes/no)**	1237 (26.93)	977 (23.29)	-3.64%	0.30
**Intermediate/Inadequate PNC**	281 (28.41)	278 (24.30)	-4.11%	0.88

*GED* General Educational Development, *WIC* Women’s, Infant’s, and Children, *BMI* body mass index

To assess the association between damage and perinatal outcomes/access to PNC services, we first categorized the percent of parcels damaged within a 25 m radius around the geocoded birth into four categories, defining all women who had births before Michael as living in areas with zero damage. Although there was higher risk of LBW and SGA in women living in the highest damaged area (Level 4) than those in women living in zero damaged area (LBW: adjusted relative risk (aRR) = 1.15, 95% CI: 0.88, 1.50; SGA: aRR = 1.14, 95%CI: 0.91, 1.41), estimates were imprecise. Moreover, the risks of having PTB, Caesarean section, and no breastfeeding after birth were not significantly different in women living in areas with different extent of damage after adjusting for confounders (Table 3). Women living in the area with relatively high damage (Level 3) were more likely to have intermediate or inadequate PNC (defined by Kotelchuck Index [24]) than women living in zero damaged areas (aRR = 1.21, 95% CI: 1.03, 1.43). The results were similar using a radius of 50 m or 100 m for damage measurement and excluding births before Michael (Supplementary Table 2).

**Table 3:** Associations between the extent of damage after Michael and perinatal outcomes and access to prenatal services (quartiles)

			**Before = 0**		**Before excluded**	
			**Unadjusted model**	**Adjusted model**^a^	**Unadjusted model**	**Adjusted model**^a^
**Low birth weight (LBW)**	**N**		680/8353	680/8353	356/3960	356/3960
	**RR (95%CI)**	**Level2:Level1**	0.949 (0.709, 1.271)	0.960 (0.717, 1.285)	0.822 (0.605, 1.117)	0.839 (0.619, 1.138)
		**Level3:Level1**	0.939 (0.648, 1.361)	0.914 (0.634, 1.318)	0.813 (0.555, 1.193)	0.799 (0.548, 1.166)
		**Level4:Level1**	1.215 (0.930, 1.588)	1.150 (0.879, 1.504)	1.052 (0.793, 1.398)	1.004 (0.756, 1.334)
**Preterm birth (PTB)**	**N**		932/7788	932/7788	434/3764	434/3764
	**RR (95%CI)**	**Level2:Level1**	1.080 (0.861, 1.355)	1.081 (0.861, 1.357)	1.122 (0.879, 1.431)	1.131 (0.887, 1.444)
		**Level3:Level1**	0.859 (0.621, 1.188)	0.842 (0.610, 1.164)	0.892 (0.647, 1.249)	0.882 (0.631, 1.233)
		**Level4:Level1**	0.828 (0.633, 1.083)	0.808 (0.617, 1.056)	0.860 (0.648, 1.141)	0.846 (0.637, 1.122)
**Small for gestational age (SGA)**	**N**		947/7788	947/7788	497/3764	497/3764
	**RR (95%CI)**	**Level2:Level1**	0.961 (0.755, 1.223)	0.971 (0.764, 1.234)	0.854 (0.663, 1.100)	0.878 (0.683, 1.129)
		**Level3:Level1**	0.912 (0.665, 1.249)	0.886 (0.651, 1.208)	0.810 (0.586, 1.121)	0.802 (0.583, 1.102)
		**Level4:Level1**	1.204 (0.965, 1.501)	1.135 (0.911, 1.414)	1.070 (0.847, 1.352)	1.025 (0.813, 1.293)
**Caesarean section**	**N**		2249/7018	2249/7018	1093/3352	1093/3352
	**RR (95%CI)**	**Level2:Level1**	0.986 (0.861, 1.130)	0.990 (0.867, 1.131)	0.959 (0.831, 1.108)	0.956 (0.830, 1.100)
		**Level3:Level1**	0.858 (0.707, 1.041)	0.872 (0.721, 1.053)	0.834 (0.683, 1.019)	0.841 (0.692, 1.022)
		**Level4:Level1**	1.098 (0.963, 1.251)	1.089 (0.956, 1.240)	1.068 (0.929, 1.227)	1.050 (0.915, 1.205)
**No breastfeeding**	**N**		1869/8207	1869/8207	895/3901	895/3901
	**RR (95%CI)**	**Level2:Level1**	0.837 (0.703, 0.996)	0.870 (0.736, 1.028)	0.824 (0.686, 0.989)	0.868 (0.728, 1.035)
		**Level3:Level1**	0.989 (0.811, 1.207)	0.937 (0.779, 1.129)	0.974 (0.792, 1.198)	0.936 (0.771, 1.135)
		**Level4:Level1**	1.154 (0.993, 1.342)	1.086 (0.943, 1.249)	1.137 (0.968, 1.335)	1,983 (0.932, 1.259)
**Gestational month of 1st PNC**	**N**		6776	6776	3219	3219
	**Difference (95%CI)**	**Level2:Level1**	0.024 (-0.125, 0.174)	0.024 (-0.125, 0.172)	-0.003 (-0.164, 0.157)	-0.005 (-0.164, 0.154)
		**Level3:Level1**	0.194 (-0.028, 0.417)	0.179 (-0.045, 0.402)	0.167 (-0.063, 0.397)	0.150 (-0.081, 0.381)
		**Level4:Level1**	0.102 (-0.064, 0.268)	0.077 (-0.088, 0.243)	0.075 (-0.101, 0.251)	0.049 (-0.127, 0.225)
**PNC (yes/no)**	**N**		8211/8330	8211/8330	3887/3958	3887/3958
	**RR (95%CI)**	**Level2:Level1**	0.999 (0.988, 1.009)	1.001 (0.990, 1.011)	1.004 (0.992, 1.015)	1.004 (0.993, 1.016)
		**Level3:Level1**	0.994 (0.979, 1.009)	0.996 (0.982, 1.011)	0.999 (0.984, 1.015)	1.000 (0.984, 1.015)
		**Level4:Level1**	0.999 (0.988, 1.010)	1.001 (0.990, 1.011)	1.004 (0.993, 1.016)	1.004 (0.992, 1.016)
**Intermediate/Inadequate PNC**	**N**		1975/7019	1975/7019	1044/3353	1044/3353
	**RR (95%CI)**	**Level2:Level1**	1.023 (0.882, 1.186)	1.007 (0.871, 1.166)	0.904 (0.774, 1.055)	0.903 (0.775, 1.052)
		**Level3:Level1**	1.255 (1.063, 1.482)	**1.211 (1.030, 1.425)**	1.109 (0.933, 1.318)	1.086 (0.917, 1.285)
		**Level4:Level1**	1.174 (1.020, 1.351)	1.124 (0.978, 1.290)	1.037 (0.895, 1.203)	1.006 (0.870, 1.165)

^a^LBW adjusting for: mother's education, age, ethnicity, smoking during pregnancy, and whether in WIC program; PTB and SGA: mother's age, education, ethnicity, pre-pregnancy BMI, smoking during pregnancy, and whether in WIC program; C-section and breastfeeding adjusting for mother's education, age, ethnicity, smoking during pregnancy, whether in WIC program, and Kotelchuck Index

We next used outcome-specific cutoffs to categorize the percent of parcels damaged within a 25 m radius around the geocoded birth into three categories (Table 4). Again, we also saw an increased risk of having LBW and SGA in the highest damaged area (Level 3) compared to women living in zero damaged areas, but the differences were small and imprecise. Women living in the highest damaged area (Level 3) were more likely to have delayed their first PNC visit (adjusted difference = 0.13 weeks, 95%CI: 0.012, 0.239) and more likely to have inadequate PNC (aRR = 1.15, 95%CI: 1.04, 1.27). Sensitivity analyses were conducted including Leon County; results were similar (Supplementary Tables 3 and 4).

**Table 4:** Associations between the extent of damage after Michael and perinatal outcomes and access to prenatal services (Outcome-specific cutoffs)

			**Before = 0**		**Before excluded**	
			**Unadjusted model**	**Adjusted model**^a^	**Unadjusted model**	**Adjusted model**^a^
**Low birth weight (LBW)**	**N**		680/8353	680/8353	356/3960	356/3960
	**RR (95%CI)**	**Level2 (0 ~ 0.14):Level1 (0)**	0.217 (0.031, 1.518)	0.253 (0.036, 1.784)	0.1881 (0.027, 1.318)	0.213 (0.030, 1.508)
		**Level3 (> = 0.14):Level1 (0)**	1.076 (0.894, 1.297)	1.046 (0.689, 1.260)	0.923 (0.757, 1.148)	0.920 (0.748, 1.131)
**Preterm birth (PTB)**	**N**		932/7788	932/7788	434/3764	434/3764
	**RR (95%CI)**	**Level2 (0 ~ 0.33):Level1 (0)**	1.093 (0.876, 1.363)	1.094 (0.878, 1.366)	1.135 (0.894, 1.440)	1.142 (0.900, 1.448)
		**Level3 (> = 0.33):Level1 (0)**	0.824 (0.663, 1.023)	**0.805 (0.648, 0.999)**	0.856 (0.677, 1.082)	0.847 (0.670, 1.070)
**Small for gestational age (SGA)**	**N**		947/7788	947/7788	497/3764	497/3764
	**RR (95%CI)**	**Level 2 (0 ~ 0.33):Level1 (0)**	0.935 (0.735, 1.188)	0.947 (0.746, 1.201)	0.831 (0.645, 1.069)	0.860 (0.670, 1.103)
		**Level3 (> = 0.33):Level1 (0)**	1.112 (0.922, 1.340)	1.057 (0.879, 1.272)	0.988 (0.808, 1.209)	0.959 (0.784, 1.172)
**Caesarean section**	**N**		1869/8207	1869/8207	895/3901	895/3901
	**RR (95%CI)**	**Level2 (0 ~ 0.33):Level1 (0)**	0.843 (0.712, 0.998)	0.880 (0.748, 1.035)	0.830 (0.694, 0.992)	0.879 (0.704, 1.043)
		**Level3 (> = 0.33):Level1 (0)**	1.092 (0.936, 1.237)	1.024 (0.911, 1.151)	1.075 (0.938, 1.233)	1.025 (0.901, 1.167)
**No breastfeeding**	**N**		2249/7018	2249/7018	1093/3352	1093/3352
	**RR (95%CI)**	**Level2 (0 ~ 0.33):Level1 (0)**	0.968 (0.846, 1.108)	0.979 (0.850, 1.108)	0.941 (0.816, 1.087)	0.941 (0.818, 1.082)
		**Level3 (> = 0.33):Level1 (0)**	1.019 (0.911, 1.140)	1.022 (0.915, 1.141)	0.991 (0.878, 1.120)	0.987 (0.876, 1.113)
**Gestational month of 1st PNC**	**N**		7324	7324	3531	3531
	**Difference (95%CI)**	**Level2 (0 ~ 0.18):Level1 (0)**	-0.165 (-0.391, 0.060)	-0.133 (-0.361, 0.095)	-0.242 (-0.475, -0.009)	-0.207 (-0.442, 0.028)
		**Level3 (> = 0.18):Level1 (0)**	0.151 (0.037, 0.265)	**0.126 (0.012, 0.239)**	0.074 (-0.055, 0.202)	0.043 (-0.086, 0.171)

^a^LBW adjusting for: mother's education, age, ethnicity, smoking during pregnancy, and whether in WIC program; PTB and SGA: mother’s age, education, ethnicity, pre-pregnancy BMI, smoking during pregnancy, and whether in WIC program; C-section and breastfeeding adjusting for mother's education, age, ethnicity, smoking during pregnancy, whether in WIC program, and Kotelchuck Index

## Discussion

In this study, we used classified residential building damage from aerial data to assess the residential patterns before and after Hurricane Michael, the change in population giving birth after Hurricane Michael, and the association between the extent of residential building damage after Hurricane Michael and adverse perinatal outcomes. Before Hurricane Michael, women with lower education and/or women of color were more likely to live in areas that would see high residential building damage compared to other impacted population groups. We found a greater proportion of births delivered by women living in the high-risk/damage area (> 25% damaged parcels after Hurricane Michael) in the year before Hurricane Michael than the year after Hurricane Michael, suggesting women giving birth were likely to have moved out of damaged areas to some extent, or that those living in the most-damaged areas had postponed pregnancy. Lastly, women living in the area with relatively high damage were more likely to have intermediate or inadequate PNC than women residing in zero damaged areas. However, we failed to identify significant associations between residential building damage after Hurricane Michael and other perinatal outcomes, including LBW, PTB, SGA, cesarean section, and breastfeeding.

Residential patterns surrounding high-risk natural disaster areas can have critical consequences for many health and economic outcomes [15]. Our study found that non-white women and women with lower education were more likely to live in high-risk areas (areas that would see high damage after Michael) before Michael. This is in line with previous findings that minority residents and lower-income people are more likely to live in high-risk flood areas [15, 27, 28]. The pattern is clear even though high-income, white residents tend to concentrate in high-risk coastal areas because of the water amenities [29]. This discrepancy may be related to properties located within high-risk flood areas being more affordable than those outside the high-risk areas. According to a study conducted in South Florida, the prices of properties located just inside a high-risk flood area are 6.3% discounted compared to those located just outside a high-risk flood area [15]. Furthermore, the National Flood Insurance Program (NFIP) might also improve the affordability of properties located within high-risk flood areas. The price for flood insurance under the NFIP, available to anyone living in the designated high-risk flood communities, is—lower than private insurances [29, 30].

Population displacement may occur after natural disasters, including people who evacuated before a disaster, returned, and migrated to adjacent states after a disaster. Before Hurricane Michael landed, 375,000 residents in Florida, Georgia and Alabama were under mandatory evacuation orders [31].Our study found that there were significantly more women who had births before Michael compared to the year after Michael (1258 (26.89%) vs. 998 (23.28%), *p* < 0.0001) in areas having high damage (> 25% damaged parcels). This suggests that women giving birth were likely to have moved out of damaged areas to some extent. The difference was significant regardless of radius considered. (Other explanations for this decline are also possible, including postponement of pregnancy and increased pregnancy losses.) Population displacement can contribute to change in perinatal outcomes after disasters. For instance, after Hurricane Katrina, women who gave birth in southern Louisiana were more likely to be non-Hispanic white, Hispanic, and better educated [1]. After a natural disaster, the evacuation and migration rates vary by race/ethnicity, homeownership, education, wealth, and age [32]. In this study, we found that women of color and less educated women giving birth were less likely to have moved out of damaged areas than White women and women with a high education level after Michael. However, the directions of the associations can be mixed over time. For example, one study shows that African Americans in New Orleans were less likely to evacuate before Hurricane Katrina compared to other populations, potentially due to a lack of adequate personal support, limited spatial networks, lack of hotel reservations, and the need to stay to collect social security payments [33]. In contrast, other studies found that African Americans in New Orleans were more likely to move out and less likely to return than white Americans in the first two years after Katrina, potentially because they were more likely to live in high damage areas and were less likely to own houses and retain their jobs [34, 35].

The association between education and evacuation/migration was also inconclusive in previous studies. People with lower education were more likely to migrate after disasters in some studies [36, 37], while other studies found that higher educated people were more likely to migrate [38, 39]. Similarly, the evidence for the association between wealth and evacuation/migration was also mixed. Some studies support the evidence that disadvantaged groups are more likely to migrate after disasters [36, 40], while other studies found the opposite [34]. In our studies, we did not have the data on household income among women giving birth. Still, we found that after the hurricane, a lower proportion of women were enrolled in the WIC program that provides health care referrals and nutritional support to low-income pregnant women, postpartum women, and children up to age five [41]. However, the decrease in enrollment in the WIC program can also be due to the reduced access to WIC services. Younger or school/working-aged people were more likely to leave after Hurricane Katrina in New Orleans [40] and Hurricane Maria in Puerto Rico [42]. Our studies did not find a significant change in maternal age at delivery before or after Hurricane Michael or in high damage and low damage areas. However, this might be due to the difference between population giving birth, usually 18–45 years old, and the general population. The conflicting evidence in previous studies might be due to the scale of the disasters, the duration and success of recovery efforts, government assistance, and the difficulty in collecting data and evaluating demographic changes after natural disasters [32]. Nevertheless, the impact of social determinants of health, such as race, socioeconomic status, and age, can be pronounced during and after disasters [42].

Previously, we used the vital statistics data and county level FEMA assistance levels as a proxy of hurricane related exposures to compare births outcomes in the year before and after Hurricane Michael in the same study area. We found that Hurricane Michael was associated with a higher risk of LBW and SGA in the year after Michael compared to the year before but using county level FEMA [43]. However, we failed to identify associations between the extent of residential building damage and the risk of adverse perinatal outcomes in the present study. There are several possible explanations for this. First, this might be due to the small sample size of this study. The fact that the estimates of risks of LBW and SGA in areas with the highest residential building damage were higher than those without damage, and the effect estimates were similar in magnitude though less precise, might support this possibility. Second, the effects of disasters on pregnancy outcome are due to a complex set of exposures, such as the stress of living in a disaster recovery environment, rather than location-specific damage. Third, regardless of damages to individual houses, damages to health facilities can reduce the accessibility to PNC for women living in the catchment of the health facilities, which can increase the risk of adverse perinatal outcomes. Previously, we found that women in the most affected area initiated PNC later and had a higher likelihood of inadequate PNC after Michael, and the delayed PNC initiation may partially explain the increased risk of SGA [6]. Finally, previous studies may have been confounded, and thus no association genuinely exists.

Strengths of this study include the household and immediate surrounding area level damage after Hurricane Michael and examination of multiple adverse perinatal outcomes. However, there are also several limitations of this study. First, although the number of births in high-risk/damage areas decreased after Hurricane Michael, we did not address a possible increase in miscarriage or reduction in fertility in those areas. This could have contributed to the lack of significant increases in risk in other pregnancy outcomes with higher damage. Second, the sample size is relatively small in each category of damage measure, which might have contributed to the null findings. Thirdly, the aerial imagery used only measured damage to rooftop a few months after landfall. Residential units could have fixed hurricane damage during the intervening period, but this is likely a small proportion due to the challenges of rebuilding and filling insurance claims post-disaster. Relatedly, rooftop damage may miss less dramatically but consequential environmental exposures that influence adverse perinatal outcomes (e.g., water damage, mold growth). Fourthly, it is possible that the address from Vital Statistics was not the address where a participant was at the time of Hurricane Michael. However, even if participants evacuated during Hurricane Michael, their house damage could still impact their pregnancy outcomes by causing stress and mental health and property/financial loss. Lastly, we were not able to control for household income in our models due to lack of information, so residual confounding is a possibility. However, maternal education might serve as a proxy for household income.

## Conclusion

Aerial imagery enables us to evaluate the impact of natural disasters on perinatal outcomes, based on an individual-level (or close to, within a house or two) measure of residential building damage. We found that women with lower education and or of Black or other non-White race/ethnicity were more likely to live in areas that would see high damage compared to other affected population groups. Moreover, women giving birth were likely to have moved out of damaged areas to some extent. Lastly, living in the area with relatively high damage increased the risk of having intermediate or inadequate PNC, but not other adverse perinatal outcomes. Future studies should evaluate whether the association between natural disasters and perinatal outcomes comparing births before and after disasters is confounded by unmeasured factors, including household income, ownership of house/flood insurance, and other environmental exposures (e.g., water damage, mold growth). Furthermore, additional impacts of natural disasters on perinatal health beyond property damage should be investigated in future studies.

## Supplementary Information

**Additional file 1:** **Supplementary Table 1.** Numbers of births in each county of residence received public and individual assistance. **Supplementary Table 2.** Associations between the extent of damage after Michael and perinatal outcomes and access to prenatal services (quartiles). **Supplementary Table 3.** Associations between the extent of damage after Michael and perinatal outcomes and access to prenatal services (quartiles), including Leon County. **Supplementary Table 4.** Associations between the extent of damage after Michael and perinatal outcomes and access to prenatal services (Outcome-specific cutoffs), including Leon County. **Supplementary Figure 1.** Number of births in each county of residence that received public and individual assistance.

## Data Availability

The datasets generated and/or analyzed during the current study are available from the Florida Department of Health’s Office of Vital Statistics upon request.
