# Supplementary material for: Remotely sensed measures of Hurricane Michael damage and adverse perinatal outcomes and access to prenatal care services in the Florida panhandle
Source: Environ Health. 2022 Nov 29;21:118. doi: 10.1186/s12940-022-00924-1 (PMC9707262; doi:10.1186/s12940-022-00924-1)
Supplement: Supplementary file 1 — Additional file 1: Supplementary Table 1. Numbers of births in each county of residence received public and individual assistance. Supplementary Table 2. Associations between the extent of damage after Michael and perinatal outcomes and access to prenatal services (quartiles). Supplementary Table 3. Associations between the extent of damage after Michael and perinatal outcomes and access to prenatal services (quartiles), including Leon County. Supplementary Table 4. Associations between the extent of damage after Michael and perinatal outcomes and access to prenatal services (Outcome-specific cutoffs), including Leon County. Supplementary Figure 1. Number of births in each county of residence that received public and individual assistance. [file 12940_2022_924_MOESM1_ESM.docx]

Supplementary tables

Table 1. Numbers of births in each county of residence received public and individual assistance

| **County** | **Before** | **After** | **Total** |
| --- | --- | --- | --- |
| BAY | 2285 | 1902 | 4187 |
| CALHOUN | 132 | 114 | 246 |
| FRANKLIN | 87 | 107 | 194 |
| GADSDEN | 499 | 504 | 1003 |
| GULF | 109 | 117 | 226 |
| HOLMES | 197 | 190 | 387 |
| JACKSON | 526 | 456 | 982 |
| LEON | 2877 | 2974 | 5851 |
| LIBERTY | 58 | 73 | 131 |
| TAYLOR | 232 | 225 | 457 |
| WAKULLA | 315 | 314 | 629 |
| WASHINGT | 238 | 285 | 523 |
| **Total** | **7555** | **7261** | **14816** |

Before: Oct.6^th^, 2017 to Oct. 5^th^; after: Oct. 6^th^ to Oct. 6^th^, 2019

Table 2. Associations between the extent of damage after Michael and perinatal outcomes and access to prenatal services (quartiles)

|  |  |  | **50 m** | | **50m** | |  | **100 m** | | **100 m** | |
| --- | --- | --- | --- | --- | --- | --- | --- | --- | --- | --- | --- |
|  |  |  | **Before=0** | | **Before excluded** | |  | **Before=0** | | **Before excluded** | |
|  |  |  | **Unadjusted model** | **Adjusted model*** | **Unadjusted model** | **Adjusted model*** |  | **Unadjusted model** | **Adjusted model*** | **Unadjusted model** | **Adjusted model*** |
| **Low birth weight (LBW)** | **N** |  | 680/8353 | 680/8353 | 356/3960 | 356/3960 |  | 680/8353 | 680/8353 | 356/3960 | 356/3960 |
|  | **RR (95%CI)** | **Level2:Level1** | 0.991 (0.756, 1.300) | 1.026 (0.784, 1.344) | 0.834 (0.623, 1.116) | 0.877 (0.657, 1.170) |  | 1.235 (0.981, 1.555) | 1.189 (0.947, 1.493) | 1.011 (0.778, 1.314) | 0.981 (0.756, 1.272) |
|  |  | **Level3:Level1** | 1.074 (0.818, 1.411) | 1.070 (0.816, 1.402) | 0.904 (0.675, 1.211) | 0.915 (0.685, 1.224) |  | 0.891 (0.688, 1.154) | 0.892 (0.689, 1.154) | 0.729 (0.547, 0.972) | 0.736 (0.553, 0.979) |
|  |  | **Level4:Level1** | 1.054 (0.814, 1.364) | 1.012 (0.781, 1.310) | 0.887 (0.671, 1.172) | 0.865 (0.655, 1.142) |  | 1.082 (0.853, 1.374) | 1.058 (0.834, 1.344) | 0.886 (0.676, 1.160) | 0.873 (0.667, 1.142) |
|  |  |  |  |  |  |  |  |  |  |  |  |
| **Preterm birth (PTB)** | **N** |  | 932/7788 | 932/7788 | 434/3764 | 434/3764 |  | 932/7788 | 932/7788 | 434/3764 | 434/3764 |
|  | **RR (95%CI)** | **Level2:Level1** | 1.026 (0.825, 1.277) | 1.045 (0.840, 1.299) | 1.050 (0.826, 1.335) | 1.083 (0.852, 1.376) |  | 1.086 (0.892, 1.323) | 1.067 (0.875, 1.299) | 1.116 (0.886, 1.405) | 1.106 (0.878, 1.392) |
|  |  | **Level3:Level1** | 0.792 ( 0.609, 1.030) | 0.784 (0.603, 1.019) | 0.811 (0.612, 1.073) | 0.813 (0.614, 1.076) |  | 0.783 (0.624, 0.981) | 0.782 (0.623, 0.981) | 0.804 (0.622, 1.038) | 0.811 (0.628, 1.047) |
|  |  | **Level4:Level1** | 0.885 (0.702, 1.116) | 0.869 (0.689, 1.096) | 0.906 (0.704, 1.166) | 0.901 (0.699, 1.159) |  | 0.898 (0.725, 1.112) | 0.886 (0.715, 1.097) | 0.922 (0.722, 1.178) | 0.919 (0.719, 1.173) |
|  |  |  |  |  |  |  |  |  |  |  |  |
| **Small for gestational age (SGA)** | **N** |  | 947/7788 | 947/7788 | 497/3764 | 497/3764 |  | 947/7788 | 947/7788 | 497/3764 | 497/3764 |
|  | **RR (95%CI)** | **Level2:Level1** | 0.922 (0.730, 1.163) | 0.947 (0.752, 1.192) | 0.801 (0.625, 1.027) | 0.844 (0.660, 1.079) |  | 1.075 (0.879, 1.316) | 1.016 (0.833, 1.239) | 0.929 (0.739, 1.166) | 0.891 (0.712, 1.115) |
|  |  | **Level3:Level1** | 1.132 (0.908, 1.410) | 1.130 (0.908, 1.405) | 0.984 (0.777, 1.247) | 1.008 (0.797, 1.274) |  | 0.958 (0.778, 1.180) | 0.950 (0.775, 1.166) | 0.827 (0.655, 1.045) | 0.833 (0.663, 1.048) |
|  |  | **Level4:Level1** | 1.066 (0.862, 1.318) | 1.022 (0.829, 1.261) | 0.927 (0.737, 1.166) | 0.911 (0.726, 1.143) |  | 1.214 (1.007, 1.464) | 1.189 (0.987, 1.433) | 1.048 (0.846, 1.299) | 1.042 (0.842, 1.291) |
|  |  |  |  |  |  |  |  |  |  |  |  |
| **Caesarean section** | **N** |  | 2249/7018 | 2249/7018 | 1093/3352 | 1093/3352 |  | 2249/7018 | 2249/7018 | 1093/3352 | 1093/3352 |
|  | **Difference (95%CI)** | **Level2:Level1** | 0.941 (0.825, 1.074) | 0.948 (0.833, 1.079) | 0.902 (0.782, 1.039) | 0.898 (0.781, 1.033) |  | 0.976 (0.865, 1.102) | 0.975 (0.866, 1.097) | 0.939 (0.811, 1.066) | 0.916 (0.802, 1.047) |
|  |  | **Level3:Level1** | 0.945 (0.820, 1.089) | 0.952 (0.830, 1.092) | 0.905 (0.778, 1.053) | 0.903 (0.780, 1.045) |  | 0.963 (0.855, 1.084) | 0.972 (0.865, 1.093) | 0.917 (0.802, 1.049) | 0.914 (0.801, 1.043) |
|  |  | **Level4:Level1** | 1.038 (0.919, 1.173) | 1.031 (0.913, 1.164) | 0.994 (0.871, 1.136) | 0.976 (0.855, 1.115) |  | 1.036 (0.923, 1.163) | 1.032 (0.920, 1.157) | 0.987 (0.866, 1.125) | 0.969 (0.851, 1.104) |
|  |  |  |  |  |  |  |  |  |  |  |  |
| **No breastfeeding** | **N** |  | 1869/8207 | 1869/8207 | 895/3901 | 895/3901 |  | 1869/8207 | 1869/8207 | 895/3901 | 895/3901 |
|  | **RR (95%CI)** | **Level2:Level1** | 0.860 (0.732, 1.010) | 0.939 (0.805, 1.096) | **0.822 (0.692, 0.978)** | 0.925 (0.784, 1.093) |  | 1.043 (0.910, 1.194) | 1.011 (0.888, 1.150) | 1.011 (0.865, 1.183) | 1.000 (0.862, 1.161) |
|  |  | **Level3:Level1** | 0.905 (0.769, 1.066) | 0.910 (0.780, 1.063) | 0.865 (0.726, 1.031) | 0.897 (0.760, 1.060) |  | 0.912 (0.792, 1.050) | 0.930 (0.814, 1.062) | 0.885 (0.753, 1.039) | 0.920 (0.790, 1.072) |
|  |  | **Level4:Level1** | 1.040 (0.903, 1.198) | 0.992 (0.869, 1.133) | 0.994 (0.852, 1.161) | 0.978 (0.845, 1.132) |  | 1.008 (0.881, 1.154) | 0.976 (0.859, 1.109) | 0.978 (0.837, 1.143) | 0.966 (0.833, 1.120) |
|  |  |  |  |  |  |  |  |  |  |  |  |
| **Gestational month of 1st PNC** | **N** |  | 6776 | 6776 | 3219 | 3219 |  | 6776 | 6776 | 3219 | 3219 |
|  | **RR (95%CI)** | **Level2:Level1** | 0.013 (-0.130, 0.156) | 0.033 (-0.109, 0.175) | -0.012 (-0.170, 0.146) | 0.011 (-0.145, 0.167) |  | 0.097 (-0.037, 0.231) | 0.102 (-0.031, 0.235) | 0.110 (-0.044, 0.264) | 0.118 (-0.035, 0.270) |
|  |  | **Level3:Level1** | 0.176 (0.011, 0.341) | **0.171 (0.007, 0.336)** | 0.151 (-0.027, 0.329) | 0.149 (-0.028, 0.326) |  | 0.141 (-0.004, 0.286) | 0.140 (-0.005, 0.285) | 0.154 (-0.009, 0.318) | 0.156 (-0.007, 0.320) |
|  |  | **Level4:Level1** | 0.086 (-0.065, 0.238) | 0.063 (-0.088, 0.214) | 0.061 (-0.104, 0.226) | 0.041 (-0.124, 0.206) |  | 0.143 (0.004, 0.283) | 0.131 (-0.006, 0.269) | 0.157 (-0.002, 0.315) | 0.148 (-0.009, 0.305) |
|  |  |  |  |  |  |  |  |  |  |  |  |
| **PNC (yes/no)** | **N** |  | 8211/8330 | 8211/8330 | 3887/3958 | 3887/3958 |  | 8211/8330 | 8211/8330 | 3887/3958 | 3887/3958 |
|  | **RR (95%CI)** | **Level2:Level1** | 0.994 (0.983, 1.005) | 0.994 (0.983, 1.005) | 1 (0.988, 1.013) | 0.998 (0.986, 1.010) |  | 0.994 (0.984, 1.004) | 0.997 (0.987, 1.007) | 0.998 (0.987, 1.011) | 0.999 (0.987, 1.011) |
|  |  | **Level3:Level1** | 1.004 (0.995, 1.013) | 1.005 (0.997, 1.014) | 1.010 (1.000, 1.021) | 1.009 (0.999, 1.019) |  | 0.986 (0.975, 0.998) | **0.987 (0.976, 0.998)** | 0.991 (0.978, 1.004) | 0.989 (0.977, 1.002) |
|  |  | **Level4:Level1** | 0.997 (0.986, 1.007) | 0.998 (0.988, 1.008) | 1.003 (0.991, 1.015) | 1.002 (0.990, 1.013) |  | 1.002 (0.994, 1.009) | 1.003 (0.995, 1.010) | 1.006 (0.996, 1.016) | 1.005 (0.995, 1.014) |
|  |  |  |  |  |  |  |  |  |  |  |  |
| **Intermediate/Inadequate PNC** | **N** |  | 1975/7019 | 1975/7019 | 1044/3353 | 1044/3353 |  | 1975/7019 | 1975/7019 | 1044/3353 | 1044/3353 |
|  | **RR (95%CI)** | **Level2:Level1** | 0.978 (0.847, 1.130) | 1.011 (0.876, 1.168) | 0.844 (0.724, 0.984) | 0.894 (0.767, 1.041) |  | 1.171 (1.036, 1.323) | 1.156 (1.024, 1.305) | 1.015 (0.883, 1.166) | 1.027 (0.896, 1.177) |
|  |  | **Level3:Level1** | 1.177 (1.026, 1.350) | **1.155 (1.010, 1.320)** | 1.015 (0.876, 1.176) | 1.021 (0.885, 1.178) |  | 1.200 (1.068, 1.349) | **1.183 (1.054, 1.328)** | 1.040 (0.910, 1.189) | 1.051 (0.922, 1.199) |
|  |  | **Level4:Level1** | 1.164 (1.025, 1.322) | 1.124 (0.991, 1.273) | 1.004 (0.875, 1.152) | 0.993 (0.867, 1.137) |  | 1.101 (0.971, 1.249) | 1.081 (0.956, 1.223) | 0.954 (0.828, 1.100) | 0.960 (0.836, 1.103) |

*LBW adjusting for: mother's education, age, ethnicity, smoking during pregnancy, and whether in WIC program; PTB and SGA: mother's age, education, ethnicity, pre-pregnancy BMI, smoking during pregnancy, and whether in WIC program; C-section and breastfeeding adjusting for mother's education, age, ethnicity, smoking during pregnancy, whether in WIC program, and Kotelchuck Index

Table 3. Associations between the extent of damage after Michael and perinatal outcomes and access to prenatal services (quartiles), including Leon County

|  |  |  | **Before=0** | |  | **Before excluded** | |
| --- | --- | --- | --- | --- | --- | --- | --- |
|  |  |  | **Unadjusted model** | **Adjusted model*** |  | **Unadjusted model** | **Adjusted model*** |
| **Low birth weight (LBW)** | **N** |  | 1230/14097 | 1230/14097 |  | 661/6905 | 661/6905 |
|  | **RR (95%CI)** | **Level2:Level1** | 0.818 (0.620, 1.081) | 0.845 (0.640, 1.115) |  | 0.721 (0.543, 0.958) | 0.766 (0.577, 1.016) |
|  |  | **Level3:Level1** | 0.855 (0.596, 1.226) | 0.849 (0.595, 1.211) |  | 0.753 (0.523, 1.085) | 0.779 (0.542, 1.117) |
|  |  | **Level4:Level1** | 1.145 (0.896, 1.462) | 1.087 (0.50, 1.390) |  | 1.008 (0.784, 1.297) | 1.001 (0.777, 1.291) |
|  |  |  |  |  |  |  |  |
| **Preterm birth (PTB)** | **N** |  | 1639/13265 | 1639/13265 |  | 816/6685 | 816/6685 |
|  | **RR (95%CI)** | **Level2:Level1** | 1.030 (0.835, 1.271) | 1.038 (0.841, 1.281) |  | 1.036 (0.834, 1.288) | 1.074 (0.864, 1.334) |
|  |  | **Level3:Level1** | 0.864 (0.635, 1.176) | 0.850 (0.626, 1.154) |  | 0.869 (0.636, 1.189) | 0.899 (0.658, 1.227) |
|  |  | **Level4:Level1** | 0.879 (0.693, 1.115) | 0.848 (0.669, 1.076) |  | 0.884 (0.693, 1.129) | 0.891 (0.697, 1.139) |
|  | **p-value** |  | 0.538 | 0.327 |  | 0.585 | 0.591 |
|  |  |  |  |  |  |  |  |
| **Small for gestational age (SGA)** | **N** |  | 1691/13265 | 1691/13265 |  | 906/6685 | 906/6685 |
|  | **RR (95%CI)** | **Level2:Level1** | 0.889 (0.710, 1.112) | 0.924 (0.739, 1.154) |  | 0.817 (0.650, 1.028) | 0.862 (0.687, 1.083) |
|  |  | **Level3:Level1** | 0.840 (0.617, 1.143) | 0.837 (0.619, 1.133) |  | 0.772 (0.565, 1.055) | 0.781 (0.575, 1.062) |
|  |  | **Level4:Level1** | 1.112 (0.905, 1.366) | 1.036 (0.844, 1.272) |  | 1.022 (0.827, 1.263) | 0.974 (0.788, 1.204) |
|  |  |  |  |  |  |  |  |
| **Caesarean section** | **N** |  | 4157/12443 | 4157/12443 |  | 2083/6198 | 2083/6198 |
|  | **RR (95%CI)** | **Level2:Level1** | 0.985 (0.874, 1.111) | 0.998 (0.887, 1.123) |  | 0.975 (0.862, 1.104) | 0.986 (0.872, 1.113) |
|  |  | **Level3:Level1** | 0.828 (0.687, 0.997) | 0.864 (0.720, 1.037) |  | 0.819 (0.679, 0.989) | 0.853 (0.709, 1.027) |
|  |  | **Level4:Level1** | 1.067 (0.948, 1.202) | 1.094 (0.973, 1.230) |  | 1.057 (0.935, 1.194) | 1.075 (0.952, 1.214) |
|  |  |  |  |  |  |  |  |
| **No breastfeeding** | **N** |  | 2870/13940 | 2870/13940 |  | 1375/6852 | 1375/6852 |
|  | **RR (95%CI)** | **Level2:Level1** | 0.913 (0.775, 1.075) | 0.912 (0.779, 1.067) |  | 0.960 (0.810, 1.137) | 0.951 (0.807, 1.119) |
|  |  | **Level3:Level1** | 1.138 (0.942, 1.376) | 1.013 (0.848, 1.209) |  | 1.197 (0.986, 1.454) | 1.056 (0.880, 1.267) |
|  |  | **Level4:Level1** | 1.289 (1.121, 1.483) | 1.098 (0.963, 1.251) |  | 1.356 (1.171, 1.570) | 1.146 (0.999, 1.316) |
|  |  |  |  |  |  |  |  |
| **Gestational month of 1st PNC** | **N** |  | 12222 | 12222 |  | 6075 | 6075 |
|  | **Difference (95%CI)** | **Level2:Level1** | 0.086 (-0.050, 0.222) | 0.059 (-0.076, 0.194) |  | 0.040 (-0.100, 0.181) | 0.006 (-0.134, 0.146) |
|  |  | **Level3:Level1** | 0.270 (0.058, 0.481) | 0.201 (-0.012, 0.415) |  | 0.224 (0.009, 0.439) | 0.151 (-0.066, 0.369) |
|  |  | **Level4:Level1** | 0.196 (0.046, 0.347) | 0.100 (-0.051, 0.250) |  | 0.151 (-0.004, 0.305) | 0.048 (-0.108, 0.204) |
|  |  |  |  |  |  |  |  |
| **No PNC** | **N** |  | 215/14074 | 215/14074 |  | 132/6903 | 132/6903 |
|  | **RR (95%CI)** | **Level2:Level1** | 0.988 (0.526, 1.857) | 0.982 (0.530, 1.821) |  | 0.748 (0.393, 1.424) | 0.814 (0.435, 1.524) |
|  |  | **Level3:Level1** | 1.418 (0.704, 2.855) | 1.295 (0.652, 2.570) |  | 1.073 (0.527, 2.185) | 1.077 (0.535, 2.166) |
|  |  | **Level4:Level1** | 0.980 (0.505, 1.903) | 0.884 (0.459, 1.705) |  | 0.742 (0.378, 1.458) | 0.722 (0.370, 1.410) |
|  |  |  |  |  |  |  |  |
| **Intermediate/Inadequate PNC** | **N** |  | 3308/12445 | 3308/12445 |  | 1808/6199 | 1808/6199 |
|  | **RR (95%CI)** | **Level2:Level1** | 1.055 (0.920, 1.211) | 1.020 (0.892, 1.168) |  | 0.958 (0.832, 1.102) | 0.916 (0.798, 1.053) |
|  |  | **Level3:Level1** | 1.304 (1.110, 1.533) | **1.206 (1.030, 1.412)** |  | 1.184 (1.004, 1.395) | 1.086 (0.925, 1.276) |
|  |  | **Level4:Level1** | 1.236 (1.087, 1.407) | 1.111 (0.978, 1.263) |  | 1.122 (0.982, 1.281) | 1.002 (0.878, 1.144) |

*LBW adjusting for: mother's education, age, ethnicity, smoking during pregnancy, and whether in WIC program; PTB and SGA: mother's age, education, ethnicity, pre-pregnancy BMI, smoking during pregnancy, and whether in WIC program; C-section and breastfeeding adjusting for mother's education, age, ethnicity, smoking during pregnancy, whether in WIC program, and Kotelchuck Index

Table 4. Associations between the extent of damage after Michael and perinatal outcomes and access to prenatal services (Outcome-specific cutoffs), including Leon County

|  |  |  | **Before=0** | |  | **Before excluded** | |
| --- | --- | --- | --- | --- | --- | --- | --- |
|  |  |  | **Unadjusted model** | **Adjusted model*** |  | **Unadjusted model** | **Adjusted model*** |
| **Low birth weight (LBW)** | **N** |  | 1230/14097 | 1230/14097 |  | 661/6905 | 661/6905 |
|  | **RR (95%CI)** | **Level2 (0~0.14):Level1 (0)** | 0.321 (0.082, 1.259) | 0.349 (0.089, 1.365) |  | 0.283 (0.072, 1.111) | 0.306 (0.078, 1.198) |
|  |  | **Level3 (>=0.14):Level1 (0)** | 0.975 (0.822, 1.157) | 0.964 (0.812, 1.144) |  | 0.859 (0.717, 1.029) | 0.883 (0.736, 1.059) |
|  |  |  |  |  |  |  |  |
| **Preterm birth (PTB)** | **N** |  | 1639/13265 | 1639/13265 |  | 816/6685 | 816/6685 |
|  | **RR (95%CI)** | **Level2 (0~0.33):Level1 (0)** | 1.051 (0.857, 1.288) | 1.058 (0.863, 1.297) |  | 1.057 (0.856, 1.306) | 1.095 (0.886. 1.352) |
|  |  | **Level3 (>=0.33):Level1 (0)** | 0.854 (0.702, 1.039) | 0.829 (0.682, 1.009) |  | 0.859 (0.701, 1.053) | 0.873 (0.711, 1.072) |
|  |  |  |  |  |  |  |  |
| **Small for gestational age (SGA)** | **N** |  | 1691/13265 | 1691/13265 |  | 906/6685 | 906/6685 |
|  | **RR (95%CI)** | **Level 2 (0~0.33):Level1 (0)** | 0.877 (0.703, 1.094) | 0.912 (0.732, 1.135) |  | 0.806 (0.642, 1.011) | 0.852 (0.680, 1.066) |
|  |  | **Level3 (>=0.33):Level1 (0)** | 1.022 (0.858, 1.218) | 0.973 (0.818, 1.159) |  | 0.940 (0.783, 1.127) | 0.913 (0.761, 1.096) |
|  |  |  |  |  |  |  |  |
| **Caesarean section** | **N** |  | 4157/12443 | 4157/12443 |  | 2083/6198 | 2083/6198 |
|  | **RR (95%CI)** | **Level2 (0~0.33):Level1 (0)** | 0.970 (0.861, 1.094) | 0.981 (0.873, 1.103) |  | 0.961 (0.849, 1.086) | 0.970 (0.859, 1.095) |
|  |  | **Level3 (>=0.33):Level1 (0)** | 0.991 (0.894, 1.098) | 1.024 (0.925, 1.133) |  | 0.981 (0.882, 1.091) | 1.007 (0.905, 1.120) |
|  |  |  |  |  |  |  |  |
| **No breastfeeding** | **N** |  | 2870/13940 | 2870/13940 |  | 1375/6852 | 1375/6852 |
|  | **RR (95%CI)** | **Level2 (0~0.33):Level1 (0)** | 0.924 (0.787, 1.084) | 0.923 (0.793, 1.076) |  | 0.972 (0.824, 1.146) | 0.963 (0.821, 1.129) |
|  |  | **Level3 (>=0.33):Level1 (0)** | 1.233 (1.097, 1.385) | 1.063 (0.953, 1.185) |  | 1.296 (1.145, 1.468) | 1.109 (0.986, 1.247) |
|  |  |  |  |  |  |  |  |
| **Gestational month of 1st PNC** | **N** |  | 12222 | 12222 |  | 6075 | 6075 |
|  | **Difference (95%CI)** | **Level2 (0~0.18):Level1 (0)** | -0.070 (-0.266, 0.126) | -0.061 (-0.259, 0.137) |  | -0.135 (-0.334, 0.064) | -0.137 (-0.338, 0.064) |
|  |  | **Level3 (>=0.18):Level1 (0)** | 0.224 (0.120, 0.328) | **0.146 (0.042, 0.250)** |  | 0.159 (0.049, 0.269) | 0.067 (-0.045, 0.179) |
|  |  |  |  |  |  |  |  |
| **NO PNC** | **N** |  | 215/14074 | 215/14074 |  | 132/6903 | 132/6903 |
|  | **RR (95%CI)** | **Level2 (0~0.29):Level1 (0)** | 0.951 (0.506, 1.788) | 0.946 (0.510, 1.754) |  | 0.720 (0.378, 1.371) | 0.787 (0.420, 1.473) |
|  |  | **Level3 (>=0.29):Level1 (0)** | 1.178 (0.720, 1.926) | 1.065 (0.655, 1.731) |  | 0.892 (0.537, 1.482) | 0.873 (0.527, 1.447) |
|  |  |  |  |  |  |  |  |
| **Intermediate/Inadequate PNC** | **N** |  | 3308/12445 | 3308/12445 |  | 1808/6199 | 1808/6199 |
|  | **RR (95%CI)** | **Level2 (0~0.22):Level1 (0)** | 0.943 (0.787, 1.131) | 0.932 (0.779, 1.116) |  | 0.856 (0.712, 1.029) | 0.837 (0.697, 1.005) |
|  |  | **Level3 (>=0.22):Level1 (0)** | 1.262 (1.149, 1.386) | **1.151 (1.050, 1.263)** |  | 1.145 (1.038, 1.264) | 1.038 (0.941, 1.145) |
|  | **p-value** | **p-value** | <0.0001 | 0.012 |  | 0.004 | 0.075 |

*LBW adjusting for: mother's education, age, ethnicity, smoking during pregnancy, and whether in WIC program; PTB and SGA: mother's age, education, ethnicity, pre-pregnancy BMI, smoking during pregnancy, and whether in WIC program; C-section and breastfeeding adjusting for mother's education, age, ethnicity, smoking during pregnancy, whether in WIC program, and Kotelchuck Index

Supplementary Figure 1. Number of births in each county of residence that received public and individual assistance.


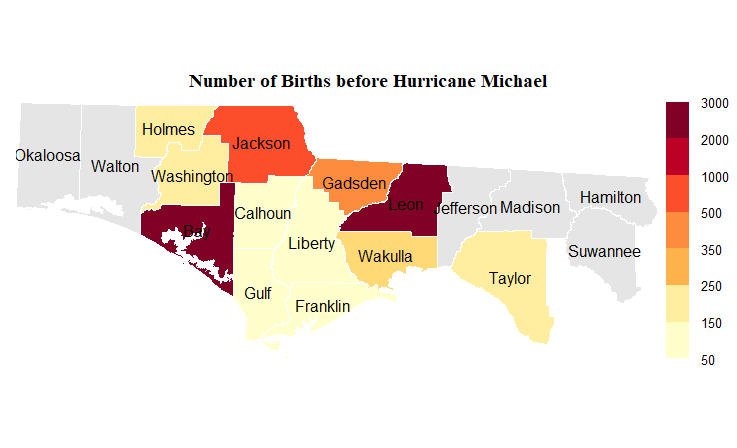

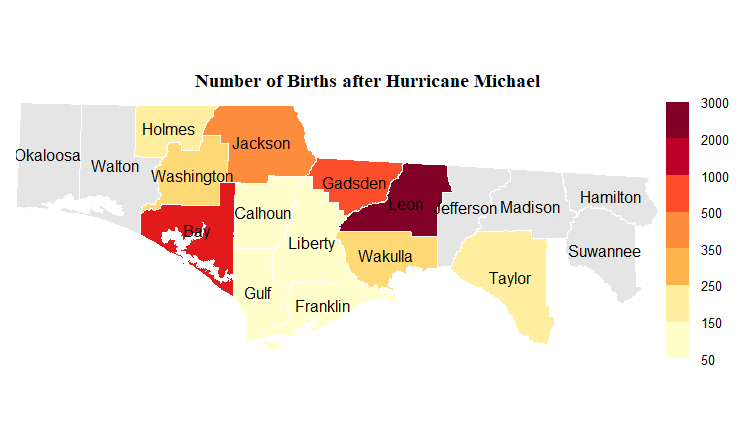


* Counties in gray received only public assistance.
